# Efficacy and toxicity of hypofractionated radiation therapy for patients with hematologic malignancies: A COVID-era ILROG collaborative report

**DOI:** 10.1016/j.radonc.2025.111200

**Published:** 2025-10-04

**Authors:** Jillian R. Gunther, Joanna C. Yang, Carla Hajj, Andrea K. Ng, Jessica L. Brady, Shuhui Cheng, Mario Levis, Shunan Qi, N. George Mikhaeel, Umberto Ricardi, Timothy M. Illidge, Anastasia Turin, Mark Knafl, Lena Specht, Bouthaina Shbib Dabaja, Joachim Yahalom, with the International Lymphoma Radiation Oncology Group

**Affiliations:** a Department of Radiation Oncology, University of Texas MD Anderson Cancer Center, Houston, TX, USA; b Department of Radiation Oncology, Washington University School of Medicine in St. Louis, USA; c Department of Radiation Oncology, Memorial Sloan Kettering Cancer Center, New York City, NY, USA; d Department of Radiation Oncology, Brigham & Women’s Hospital, Boston, MA, USA; e Department of Clinical Oncology and Radiotherapy, Guy’s Cancer Centre, Guy’s and St Thomas’ Hospital, London, United Kingdom; f Division of Cancer Sciences, Medical and Health Sciences University of Manchester, United Kingdom; g Department of Oncology, University of Torino, Torino, Italy; h Department of Radiation Oncology, Cancer Hospital, Chinese Academy of Medical Sciences and Peking Union Medical College, Beijing, China; i Division of Cancer Sciences, University of Manchester, NIHR Manchester BRC, The Christie NHS Foundation Trust, United Kingdom; j Palantir Technologies, Palo Alto, CA, USA; k Department of Genomic Medicine, University of Texas MD Anderson Cancer Center, Houston, TX, USA; l Department of Oncology, Rigshospitalet, Copenhagen University Hospital, Copenhagen, Denmark

**Keywords:** Hypofractionation, Radiation Therapy, Hematologic Malignancies, COVID-19

## Abstract

**Background and purpose:**

During the COVID19 pandemic, shorter radiation therapy (RT) courses were needed to minimize patient exposure, ensure staff safety, and conserve healthcare resources. In response, guidelines were published by the International Lymphoma Radiation Oncology Group (ILROG) to guide treatment of hematologic malignancies patients with hypofractionated radiation therapy (hRT) regimens. However, outcomes for these hypofractionated dose/fractionation regimens in terms of efficacy and toxicity are unknown.

**Materials and methods:**

In collaboration with ILROG, we performed a retrospective multinational, multicenter study. We included patients treated from 01 January 2020 to 01 September 2020 with hRT given according to the published ILROG guidelines or hRT given at > 3 Gy per fraction. We abstracted patient and treatment data from institutional databases. CTCAE v5.0 was used to grade toxicity.

**Results:**

We included 219 patients from 8 different institutions treated with 255 RT courses. Median RT dose was 12 Gy (range 4–39) in a median of 3 fractions (range 1–13). Median follow up was 232 days, with 151 patients (69 %) alive at last follow up. Response within the RT field was assessed in 210 sites, and 127 sites (60 %) had a confirmed complete response. Maximal toxicities (per site) reported were Grade 1 (n = 48), Grade 2 (n = 25), Grade 3 (n = 3) and Grade 4 (n = 1). Grade 3/4 toxicities included dermatitis, pain, and hematologic toxicity.

**Conclusions:**

Treatment of hematologic malignancies patients with hRT was generally well tolerated with few unexpected toxicities. These data provide guidance for emergencies, and hRT may be useful and could be considered even in routine settings, especially for certain patient subgroups.

## Introduction

While many malignancies are now routinely treated safely and efficaciously with hypofractionated radiation courses, hematologic malignancies have long relied on conventional fractionation with little to no data for hypofractionation. At the start of the COVID19 pandemic, cancer patients were quickly identified as high-risk for serious complications from COVID19 infection due to immunosuppression from disease and therapy. Patients with hematologic malignancies (HM) were felt to be even more vulnerable than others with cancer due to the prevalence of immunosuppressive and B-cell directed systemic therapy (ST) comprising common treatment regimens. As such, recommendations for treatment modifications were quickly developed to keep HM patients as safe as possible. Guidelines for the overall care of patients with HM, focused mainly on systemic therapy administration, were developed and published[1,2]. Treatment plans were modified to reduce or omit ST to avoid the commonly associated cytopenias and immunosuppression. In this way, RT became increasingly utilized as a bridge therapy until full, standard systemic therapy regimens could be given in a safe manner.

It became quickly evident that shorter radiation therapy (RT) courses were necessary to minimize patient exposure, ensure staff safety, and conserve healthcare resources. Hypofractionated (HF) RT is the use of higher doses, i.e. > 2 Gy, per fraction to deliver a biologically equivalent dose over a shorter number of treatment days. These shorter regimens had already been widely adopted as common treatment regimens for diseases such as breast[3] and prostate cancer[4], but data to guide the use of HF RT for HM patients was not available. The prior lack of enthusiasm for HF RT in HM patient populations likely related to the concerns for increased late toxicities due to the larger radiation doses per day, particularly considering the high probablity of cure for many HM patients. Also, given the radiosensitivity of most HMs, HF RT was not necessary to achieve excellent local control rates.

HMs, in general, exhibit very little repair of radiation damage; hence, giving the treatment in larger fractions may not increase the biologic effect on the tumor cellsand may only result in an increase in long-term damage to normal tissues. For this reason, hypofractionation has historically been avoided in HMs. However, modern highly conformal radiation techniques with steep dose gradients around the target lead to much lower doses to the surrounding normal structures, hence, even with larger fractions to the target, only the very closest parts of the normal tissues will receive fractions of > 2 Gy. The implementation of modern highly conformal RT techniques therefore opens up the possibility of using hypofractionation without significant increase in long-term toxicity.

In response to this worldwide emergency, a guideline was published by the International Lymphoma Radiation Oncology Group (ILROG)[5] to guide the treatment of HM patients with hypofractionated radiation therapy regimens. These guidelines suggested dose/fractionation schemes based on the known radiosensitivity of various HMs with consideration of the biologically equivalent doses in the HF setting. Recommendations were differentiated for patients with or without significant cardiac/lung exposure and overlapping critical structures. Since that time, additional guidelines have been created by countries and organizations to guide the use of RT during emergencies such as the pandemic, most focused on general radiation oncology and all malignancies [6–10].

Although derived from expert consensus, efficacy and toxicity data for the stated dose/fractionation schemes were not available at the time of publication. To address this deficiency, we carried out a multi-center, multi-national retrospective study examining the outcomes for HM patients treated with HF RT regimens during the earliest period of the COVID19 pandemic.

## Methods

### Patients

In collaboration with the International Lymphoma Radiation Oncology Group (ILROG), we performed a multinational, multi-institutional retrospective study including 8 centers. We secured individual institutional review board approval (or equivalent) at each participating institution. Data including patient, disease, and treatment/response characteristics (Tables 1 and 2) were abstracted from individual institutions. A Data Dictionary was given to each participating institution to guide data abstraction and coding. All data was submitted to a single REDCap database[11] according to an approved protocol.

For MDACC patient data, electronic health data were obtained from structured and unstructured electronic health record elements within the Context Engine, MD Anderson’s data management system. All data were integrated using Palantir Foundry (Syntropy), an interoperable and extensible cloud-based platform within the Context Engine that streamlines back-end data management and front-end data analysis, allowing rapid insight from multi-sourced data streams.

All patients treated from 01 January 2020 to 01 September 2020 with hRT given according to the published ILROG guidelines[5] or hRT given at > 3 Gy per fraction were included.

Patients were staged using the Lugano Staging System and Hodgkin Risk Group (if applicable) was defined as early favorable, early unfavorable, or advanced per German Hodgkin Study Group definitions[12].

### Treatment details

Both systemic therapy (ST) and RT details were recorded. Treatment intents were defined as follows: definitive, RT given alone with curative intent; consolidative, therapy given in the setting of systemic therapy with complete response; salvage, RT used to treat progressive disease when systemic therapy has failed or cannot be given/more aggressive dose intended to eradicate disease within RT field; palliative, RT given with intent to relieve symptoms, not necessarily long-term disease control; bridging for CAR T-cell therapy, RT used specifically prior to CAR T-cell therapy for the purpose of disease debulking or palliation. RT dose and technique were at the discretion of the treating physician. RT techniques included 2-dimensional/electron-beam, 3-dimensional conformal (3D-CRT), and intensity-modulated (IMRT). Systemic therapy included cytotoxic chemotherapy, targeted therapy, immunotherapy/cellular therapy. Steroids alone were not included. Treating physicians determined if a patient would be classified as having treatment with an “Alternative Schedule.” Concurrent ST was defined as ST given any time during the RT course with overlapping dates. Treatment sites were categorized as head and neck, thorax, abdomen, pelvis, extremity, and central nervous system (including brain and spine). Cutaneous sites were included in these categories. Treatments were defined as “low dose” for treatments with equivalent dose (EQD2_α/β=3_) < 20 Gy and “high dose” for treatments with EQD2_α/β=3_ ≥ 20 Gy.

### Outcome assessment

Follow up schedules and response assessment (clinical, imaging, endoscopy, etc.) were determined by the treating physician. Response was assessed using the Lugano Criteria[13]. If clinical exam was used for assessment, each physician assessed whether response was most consistent with complete response (CR), partial response (PR), stable disease (SD) or progressive disease (PD). First response in RT field, relapse in RT field, local control within RT field at one year from start of RT, and CR status at any timepoint were recorded. Variables including age, sex, systemic therapy, and ECOG are reported per patient; else, all details are reported per treated site.

### Toxicity assessment

Toxicities were graded prospectively by treating physicians or retrospectively using clinical documentation and the National Cancer Institute Common Terminology Criteria for Adverse Events (version 5.0). Acute toxicity was defined as symptoms noted within 90 days of RT end date. Late toxicities were noted if occurring after 90 days from RT end date. Maximal toxicities were reported per site.

### Statistical analyses

Univariable analyses were performed to assess relationships between clinical factors (dose characteristics, demographics, patient and disease characteristics) and patient outcomes (relapse and toxicity) using the Code Workbooks feature of the Foundry platform with R and Python code.[14] Differences between groups were assessed with χ^2^, Fisher exact, and t-tests. *P* values <=.05 were considered significant. Treatment day savings were calculated by comparing the HF and standard fraction numbers as per the ILROG guidelines[5].

## Results

We included 219 patients from 8 different institutions treated with 255 RT courses. Patient and treatment details are reported in Tables 1 and 2. Median patient age was 68 years (range 24–94) with an ECOG status of 1 at time of RT. The most common diagnoses including multiple myeloma (n = 61, 24 %), diffuse large B cell lymphoma (n = 53, 21 %), and Grade 1–2 follicular lymphoma (33, 13 %). Most sites were treated with palliative intent therapy (n = 156, 61 %). Prior systemic therapy was given to 137 patients; of those, 49 patients had received greater than three prior regimens (22 % of the total patient cohort). Of 137 patients with prior ST and response assessments, 75 patients (55 %) had progressive disease at the time of RT.

Practitioners reported choosing an alternative RT schedule due to: desire to decrease patient exposure to hospital (90, 35 %), COVID19 positive patient status (1, 0.1 %), staffing issues alone (0, 0 %), both patient exposure and staffing issues (153, 60 %), or other (11, 4 %). Median RT dose was 12 Gy (range 4–39) in a median of 3 fractions (range 1–13). The most common dose/fractionate scheme was 4 Gy in 1 fraction (74 treatments, 29 %) followed by 20 Gy in 5 fractions (42 treatments, 16 %). Nineteen treatments (7 %) were ≥ 30 Gy. The median doses for follicular lymphoma (Gr1-2) and DLBCL were 4 Gy (1 fx) and 25 Gy (5 fx), respectively. Two patients (1 %) had treatment breaks of greater than 5 days during their planned RT. Twenty-four patients (10 %) with 33 treated sites (13 %) had received prior RT in the treatment field (re-irradiation). Sixteen patients (7 %) with 18 treated sites (7 %) received concurrent ST.

Median follow up was 232 days (range 0–1752), with 151 patients (69 %) alive at last follow up. Response within the RT field was assessed in 210 sites: complete response (CR) (n = 127, 60 %); partial response (PR) (n = 58, 28 %); stable disease (SD) (n = 14, 7 %); and progressive disease (PD) (n = 11, 5 %). At one year, 71 patients with 82 sites had response assessments and disease responses within the RT field (by treated site) were consistent with a: CR (n = 60, 73 %); PR (n = 9, 11 %); SD (n = 1, 1 %); and PD (n = 12, 15 %). Of the 148 patients without response assessment at 1 year, 52 patients were dead and 96 were lost to follow up. 135 patients (62 %) achieved a CR in the RT field at some point during follow up. 16 patients (7 %) with 19 sites (7 %) relapsed within the RT field. Disease relapses were associated with prior RT in field (p = 0.004) and palliative-intent RT (p = 0.02) (Table 3).

Maximal toxicities reported were Grade 1 (n = 48), Grade 2 (n = 25), Grade 3 (n = 3) and Grade 4 (n = 1). Factors associated with any toxicity included higher RT dose (p < 0.001) and fraction number (p < 0.001) (Table 4); however, these factors were not significantly associated with Grade 3 or greater toxicity (p = 0.45 and p = 0.66, respectively). Of patients who experienced any toxicity (vs. none), more were treated with consolidative treatment intent (17.9 % vs. 6.8 %, p = 0.014). Grade 3 toxicities occurred with the following treatment regimens: 12 Gy in 3 fx to extremity (dermatitis, given concurrent with methotrexate); 30 Gy in 6 fx to extremity (dermatitis); 20 Gy in 5 fx to pelvis (pain). One grade 4 toxicity occurred with 12 Gy in 3 fx to the CNS (hematologic toxicity). With follow up of over three years, the patient has not experienced any late neurotoxicity. Details of the Grade 3 and 4 toxicities are reported in Table 5.

With hypofractionation, there were 934 treatment days, compared to an expected 2042 treatment days if the patients were treated with standard fractionation. Hypofractionation saved a total of 1108 fractions or patient visits.

## Discussion

In this study, we found that HF RT for HM patients resulted in reasonable efficacy and toxicity profiles. This is the largest study, to our knowledge, to report outcomes for a heterogeneous group of HM diagnoses treated with HF RT. Certain dose and fractionation schemes (e. g. 4 Gy in 1 fraction) were adopted more commonly, and certain patient populations were more likely to be treated with HF RT regimens. We found low rates of Grade 3 or higher toxicities, even in this diverse patient population where many had received significant prior therapy or concurrent systemic therapy. This may be related to the differentiation proposed in the ILROG guidelines of patients with or without significant cardiac/lung exposure and overlapping critical structures. Response rates in terms of local control were also encouraging.

Compared to solid malignancies, HF for HM patients has previously been minimally explored. One recent study examined outcomes for 36 HM patients treated with HF RT during the COVID era and found excellent local control rates.[15] Aggressive non-Hodgkin lymphoma patients, typically older and with poorer prognosis, have been treated with HF regimens. One such study showed no difference in response or local progression compared to conventional fractionation. OS, worse with HF, was likely due to the poorer prognosis of this group chosen to receive HF RT [16]. In a single institution study, authors found no difference in OR, time to local failure or OS for DLBCL patients treated with HF vs. SF salvage or palliative-intent RT;[17] they comment that higher RT dose (EQD2 ≥ 35 Gy) might improve outcomes. In early experiences utilizing HRT regimens such as 20 Gy in 5 fractions for bridging treatment prior to CAR T cell therapy, [18,19] most reported toxicities were low grade.

For cutaneous T cell lymphoma (CTCL) patients, a prospective trial evaluated ultra hypofractionated low dose total skin electron beam therapy (TSEBT) (8 Gy in 2 fractions)[20] and reported this as a safe and feasible alternative to SF. Another group studied HF (defined as ≥ 2.5 Gy per fraction) TSEBT for CTCL patients and concluded that these regimens provide satisfactory response and toxicity profiles.[21].

Oftentimes, plasmacytoma involves the spine vertebral bodies, and HF regimens similar to those used for solid spine metastases have been explored. A prospective trial of stereotactic body radiotherapy (SBRT) for bone plasmacytomas evaluated outcomes after SBRT regimens such as 24 Gy in 3 fractions or 30 Gy in 5 fractions; no patients has local progression although two had progression to multiple myeloma.[22] There were no grade 3 toxicities. Spine stereotactic radiosurgery (14–16 Gy in a single fraction) in treatment for multiple myeloma was analyzed and found to impart rapid and durable symptomatic responses with high local control rates of 91 % at 12 months.[23] The ILROG guidelines for plasmacytoma recommend different dose/fractionation regimens, some HF, dependent upon the scenario.[24].

From the above discussion, it is evident that HF RT regimens have thus far been explored and adopted in older patients with poorer prognosis. This is likely due to concerns for HF-induced late toxicity. In our study, we also observed lower utilization of the HF regimens in younger patients with more favorable/curable diagnoses. Similarly, other studies have found high compliance to pandemic-adapted RT guidelines except for younger patients[25]. As HF regimens have been widely adopted in breast [3] and prostate [4] cancer, long term data from the younger patients in these populations may provide reassurance that HF regimens are safe. Given the expected excellent prognosis of pediatric and adolescent and young adult (AYA) patients with curable diagnoses such as Hodgkin lymphoma, it is less likely that HF regimens will be readily adopted. There were also few patients treated with brain or craniospinal irradiation using HF regimens, possibly due to concern for late neurotoxicity, which is affected by both total dose and dose per fraction. [26,27] Additionally, many patients requiring brain RT for HM have already received central nervous system-directed chemotherapies (e.g methotrexate, cytarabine) with their own associated neurotoxicity profiles.

Unsurprisingly, we observed that patients experienced toxicity more often with increasing dose and consolidative RT, which is typically given with curative intent. We observed few Grade 3 or greater toxicities in our patient population. Only one of these patients had concurrent systemic therapy which was attributed as the reason for the severe dermatitis. Pain, experienced by one of the patients, is often difficult to predict in patients receiving RT. Hematologic toxicity is expected after craniospinal irradiation, but the timing and severity is unknown after HF and further attention to this possible complication in the setting of HF is warranted. It is reassuring that the patient treated with hypofractionated craniospinal treatment has not experienced late neurotoxicity, one of the major concerns with HF RT in the CNS.

Importantly, these HF regimens could be considered not only in times of emergency, as there are several advantages to HF RT. Patients benefit from reduced time on treatment and often reduced costs associated with more protracted RT courses. We observed a reduction in treatment days of approximately 54 %, which would translate to a significant cost savings. Additionally, ST is sometimes held during RT due to concerns for increased toxicity; HF RT regimens would allow for less time off of systemic treatment. With the increased utilization of CD19-directed CAR T-cell therapy, in which there is a built in time period during which CAR manufacturing occurs, these hypofractionated regimens may be applicable to bridging radiation therapy. It is important that practitioners have a sense of the most appropriate clinical scenarios in which HF RT can be incorporated. The patient age, prognosis, concurrent systemic therapies, and site of treatment are just a few of the factors that should be considered.

In addition to the more obvious logistical advantages of HF RT, there may be additional clinical benefits to these regimens. HF RT can reduce the risk of lymphopenia[28], which is important to allow patients to receive further systemic therapy or undergo successful apheresis for procedures such as autologous stem cell transplant or CART cell therapy. Early work has also demonstrated that HF RT can potentially create a more favorable anti-tumor immune response to augment the outcomes effected by RT alone.[29].

Our study is limited by the heterogeneous patient population and, therefore, small numbers in each patient group that preclude drawing definitive conclusions about each treatment regimen. In this retrospective study, toxicities were limited to those documented in the chart. Also, the outcomes and toxicities for each of these regimens were not compared directly with the SF regimen in a prospective manner. However, given the number of HM diagnoses and rarity of many of these, the data collected are still valuable and will serve as a basis for future evaluations.

In conclusion, our study evaluated HM patients who were treated with HF RT regimens during the earliest period of the COVID19 pandemic. We now have data to support the expert-derived HF guidelines and reassurance that the outcomes and toxicity profiles of these regimens appear reasonable. We eagerly await the results of additional studies that continue to evaluate these novel dose and fractionation regimens for our unique HM patient population.

## Figures and Tables

**Table 1:** Patient Characteristics.

	All Doses	Low Doses	High Dose
	N (%)	N (%)	N (%)
Age in years at RT start (median, range)	68 (24–94)		
Sex			
Male	103 (47)		
Female	116 (53)		
ECOG (median, range)	1 (0–4)		
Diagnosis (reported by sites treated)	255	136 (53)	119 (47)
Classic Hodgkin Lymphoma	5 (2)	0 (0)	5 (100)
DLBCL: Diffuse Large B Cell	53 (21)	3 (6)	50 (94)
Mantle Cell	10 (4)	8 (80)	2 (20)
Marginal Zone	24 (9)	21 (88)	3 (12)
Follicular Grade 1–2	33 (13)	26 (79)	7 (21)
Follicular Grade 3 (A and 3B)	4 (2)	3 (75)	1 (25)
Multiple Myeloma	61 (24)	26 (43)	35 (57)
Solitary Plasmacytoma	3 (1)	0 (0)	3 (100)
Mycosis Fungoides	18 (7)	18 (100)	0 (0)
Peripheral T cell	2 (1)	2 (100)	0 (0)
Nodular Lymphocyte Predominant	1 (0.3)	0 (0)	1 (100)
Hodgkin Lymphoma			
Leukemia (including myeloid sarcoma)	12 (5)	11 (92)	1 (8)
Other	29 (11)	18 (62)	11 (38)

**Table 2:** Treatment Characteristics for each RT site.

	All Doses	Low Dose	High Dose
	N (%)	N (%)	N (%)
Therapy Intent	255	136 (53)	119 (47)
Definitive	51 (20)	30 (59)	21 (41)
Consolidative	26 (10)	1 (4)	25 (96)
Salvage	12 (5)	3 (25)	9 (75)
Palliative	156 (61)	102 (65)	54 (35)
Bridge to CAR T-cell therapy	8 (3)	0 (0)	8 (100)
Unknown	2 (1)	0 (0)	2 (100)
If prior systemic therapy (ST)	N = 149	55 (37)	94 (63)
Prior ST regimens > 3	58 (39)	21 (36)	37 (64)
Response to ST			
CR	33 (22)	9 (27)	24 (73)
PR	28 (19)	7 (25)	21 (75)
SD	2 (1)	1 (50)	1 (50)
PD	86 (58)	38 (44)	48 (56)
Unknown	0 (0)	0 (0)	0 (0)
Treatment site			
Head and neck	47 (18)	25 (53)	22 (47)
Thorax	61 (24)	32 (52)	29 (48)
Abdomen	13 (5)	6 (46)	7 (54)
Pelvis	57 (22)	32 (56)	25 (44)
Extremity	49 (19)	26 (53)	23 (47)
Central Nervous System	26 (10)	15 (58)	11 (42)
Other	2 (1)	0 (0)	2 (100)
Radiation Dose in Gy (median, range)	12 (4–39)	4 (4–14)	20 (16–39)
Fractions (median, range)	3 (1–13)	1 (1–4)	5 (4–13)
Fraction size			
3 Gy	34 (13)	1 (3 %)	33 (97 %)
3.5 Gy	16 (6)	10 (63 %)	6 (37 %)
4 Gy	158 (62)	99 (63 %)	59 (37 %)
5 Gy	22 (9)	1 (5 %)	21 (95 %)
8 Gy	25 (10)	25 (100 %)	0 (0 %)
Dose/Fractionation			
4 Gy / 1 fx	74 (29)	74 (29)	
7 Gy / 2fxs	1 (0.3)	1 (0.3)	
8 Gy / 1 fx	25 (10)	25 (10)	
8 Gy / 2 fxs	10 (4)	10 (4)	
9 Gy / 3 fxs	1 (0.3)	1 (0.3)	
10 Gy / 2 fxs	1 (0.3)	1 (0.3)	
10.5 Gy / 3 fxs	7 (3)	7 (3)	
12 Gy / 3 fxs	15 (6)	15 (6)	
14 Gy / 4 fxs	2 (1)	2 (1)	
16 Gy / 4 fxs	17 (7)		17 (7)
17.5 Gy / 5 fxs	6 (2)		6 (2)
18 Gy / 6 fxs	2 (1)		2 (1)
20 Gy / 5 fxs;	42 (16)		42 (16)
25 Gy / 5 fxs;	11 (4)		11 (4)
27 Gy / 9 fxs;	22 (9)		22 (9)
30 Gy / 6 fxs;	10 (4)		10 (4)
36 Gy / 12 fxs;	7 (3)		7 (3)
39 Gy / 13 fxs;	2 (1)		2 (1)
Prior RT received in field (n = 253)	33 (13)	19 (58)	14 (42)
Concurrent ST (n = 254)	18 (7)	12 (67)	6 (33)

**Table 3:** Factors associated with relapse in RT field.

	All Doses			Low Dose			High Dose		
	Relapse N = 19	No Relapse N = 236	P value	Relapse N = 12	No Relapse N = 124	P value	Relapse N = 7	No Relapse N = 112	P value
**RT Dose (Gy)** Median (range)	12(4, 27)	12(4, 39)	0.16	4(4, 12)	4(4, 14)	0<.001	20(16, 27)	20(16, 39)	0.20
**Fraction Number** Median (range)	3(1, 9)	3(1, 13)	0.16	1(1, 3)	1(1, 4)	0.91	5(4, 9)	5(4, 13)	0.22
**Prior RT in Field**			0.004			0.12		0.04	
No	12 (5 %)	208 (95 %)		8 (7 %)	107 (93 %)		101 (96 %)	4 (4 %)	
Yes	7 (21 %)	26 (79 %)		4 (21 %)	15 (79 %)		11 (79 %)	3 (21 %)	
Unknown	0 (0 %)	2 (100 %)		0 (0 %)	2 (100 %)		0 (0.0 %)	0 (0.0 %)	
**Concurrent Systemic Therapy**			0.89			1.00			0.79
Yes	2 (11 %)	16 (89 %)		1 (8 %)	11 (92 %)		1 (17 %)	5 (83 %)	
No	17 (7 %)	219 (93 %)		11 (9 %)	112 (91 %)		6 (5 %)	107 (95 %)	
Unknown	0 (0 %)	1 (100 %)		0 (0 %)	0 (0 %)		0 (0 %)	0 (0 %)	
**Treatment Site**			0.64			0.61			0.69
Head/neck	3 (6 %)	44 (94 %)		2 (8 %)	23 (92 %)		1 (5 %)	21 (95 %)	
Thorax	4 (7 %)	57 (93 %)		3 (9 %)	29 (91 %)		1 (3 %)	28 (97 %)	
Abdomen	2 (15 %)	11 (85 %)		1 (17 %)	5 (83 %)		1 (14 %)	6 (86 %)	
Pelvis	5 (9 %)	52 (91 %)		2 (6 %)	30 (94 %)		3 (12 %)	22 (88 %)	
Extremity	5 (10 %)	44 (90 %)		4 (15 %)	22 (85 %)		1 (4 %)	22 (96 %)	
CNS	0 (0 %)	26 (100 %)		0 (0 %)	15 (100 %)		0 (0 %)	11 (100 %)	
Other	0 (0 %)	2 (100 %)		0 (0 %)	0 (0 %)		0 (0 %)	0 (0 %)	
**Treatment Intent**									
Definitive	0 (0 %)	51 (100 %)	0.05	0 (0.0 %)	30 (100 %)	0.12	0 (0.0 %)	21 (100 %)	0.45
Consolidative	1 (4 %)	25 (96 %)	0.72	0 (0 %)	1 (100 %)	1.00	1 (5 %)	24 (95 %)	1.00
Salvage	1 (8 %)	11 (92 %)	0.99	1 (33 %)	2 (67 %)	0.63	0 (0 %)	9 (100 %)	0.97
Palliative	17 (11 %)	139 (89 %)	0.02	11 (11 %)	91 (89 %)	0.30	6 (11 %)	48 (89 %)	0.07
Bridging for CAR T-cell therapy	0 (0 %)	8 (100 %)	0.89	0 (0 %)	0 (0 %)		0 (0 %)	8 (100 %)	1.00

**Table 4:** Factors associated with toxicity.

	All Dose						Low Dose			High Dose		
	Toxicity ≥ 3	No Toxicity ≥ 3	P value	Any toxicity	No toxicity	P value	Any toxicity	No toxicity	P value	Any toxicity	No toxicity	P value
**Per Patient**												
**Age at RT (yrs)**	N = 4	N = 215		N = 67	N = 152		N = 15	N = 98		N = 53	N = 55	
Median (range)	58 (53–77)	68 (24–100)	0.52	64 (24–93)	68 (31–94)	0.26	72.0 [41.0, 85.0]	68.0 [31.0, 94.0]	0.78	64 [24.0, 93.0]	67.0 [36.0, 82.0]	0.40
**ECOG**	N = 4	N = 211		N = 65	N = 150		N = 15	N = 98		N = 51	N = 53	
Median [Min, Max]	0 (0–2)	1 (0–4)	0.34	1.00 [0, 3.00]	1.00 [0, 4.00]	0.10	1.00 [0, 2.00]	1.00 [0, 4.00]	0.27	1.00 [0, 3.00]	1.00 [0, 4.00]	0.29
**Concurrent Systemic Therapy**	N = 4	N = 215		N = 67	N = 152		N = 15	N = 98	0.36	N = 53	N = 55	0.34
Yes	0 (0 %)	16 (100 %)	0.99	4 (25 %)	12 (75 %)	0.81	0 (0 %)	11 (100 %)		4 (80 %)	1 (20 %)	
No	4 (2 %)	198 (98 %)		63 (31 %)	139 (69 %)		15 (15 %)	86 (85 %)		49 (48 %)	54 (52 %)	
Unknown	0 (0 %)	1 (100 %)		0 (0 %)	1 (100 %)		0 (0 %)	1 (100 %)		0 (0 %)	0 (0 %)	
**Per Treatment Site**												
**RT Dose Gy** Median (range)	12(12, 30)	12(12, 39)	0.45	20(4, 39)	8(4, 36)	<0.001	4(4, 14)	4(4, 14)	0.34	25(16,39)	20(16, 36)	0.09
**Fraction Number** Median (range)	3(3, 6)	3(1, 13)	0.66	5(1,13)	2(1, 12)	<0.001	1(1, 4)	1(1, 4)	0.15	5(4, 13)	5(4, 12)	0.02
**CTV Volume**	N = 2	N = 139		N = 44	N = 97		N = 13	N = 61		N = 31	N = 36	
Median in ccs (range)	1340 (719, 1960)	180 [0.800, 4100]	0.37	234 (10.8, 3910)	143 [0.800, 4100]	0.26	511 (36.0, 3910)	126 [0.800, 4100]	0.09	200 (10.8, 2470)	255 [13.8, 1910]	0.69
**Prior RT in field**	N = 5	N = 248		N = 78	N = 177	0.35	N = 19	N = 115	0.57	N = 59	N = 60	0.38
Yes	2 (7 %)	31 (93 %)	0.26	13 (43 %)	20 (57 %)		4 (21 %)	15 (79 %)		5 (36 %)	9 (64 %)	
No	3 (1 %)	217 (99 %)		65 (30 %)	155 (70 %o)		15 (13 %)	100 (87 %)		55 (52 %)	50 (48 %)	
Unknown	0 (0 %)	2 (100 %)		0 (0 %)	2 (100 %)		0 (0 %)	2 (100 %)		0 (0 %)	0 (0 %)	
**Treatment Site**	N = 5	N = 250	0.29	N = 78	N = 177	0.61	N = 19	N = 117	0.77	N = 59	N = 60	0.62
Head/neck	0 (0 %)	47 (100 %)		18 (38 %)	29 (62 %)		5 (20 %)	20 (80 %)		13 (59 %)	9 (41 %)	
Thorax	0 (0 %)	61 (100 %)		21 (34 %)	40 (66 %)		5 (16 %)	27 (84 %)		16 (55 %)	13 (45 %)	
Abdomen	0 (0 %)	13 (100 %)		4 (31 %)	9 (69 %)		0 (0 %)	6 (100 %)		4 (57 %)	3 (43 %)	
Pelvis	1(2 %)	56 (98 %)		16 (28 %)	41 (72 %)		4 (13 %)	28 (87 %)		12 (48 %)	13 (52 %)	
Extremity	3 (6 %)	47 (94 %)		14 (29 %)	35 (71 %)		4 (16 %)	22 (84 %)		10 (43 %)	13 (57 %)	
CNS	1 (%)	25 (%)		5 (%)	21 (%)		1 (%)	14 (%)		4 (%)	7 (%)	
Other	0 (0 %)	2 (100 %)		0 (0 %)	2 (100 %)		0 (0 %)	0 (0 %)		0 (0 %)	2 (100 %)	
Unknown	0 (0 %)	0 (0 %)		0 (0 %)	0 (0 %)		0 (0 %)	0 (0 %)		0 (0 %)	0 (0 %)	
**Treatment Intent**	N = 5	N = 250	0.7	N = 78	N = 177	0.03	N = 19	N = 117	0.84	N = 59	N = 60	0.2
Definitive	0 (0 %)	51 (100 %)	0.57	15 (29 %)	36 (71 %)	0.94	5 (17 %)	25 (83 %)	0.85	10 (48 %)	11 (52 %)	1.00
Consolidative	1 (4 %)	25 (96 %)	0.99	14 (54 %)	12 (46 %)	0.01	0 (0.0 %)	1 (100 %)	1.00	14 (56 %)	11 (44 %)	0.62
Salvage	0 (0 %)	12 (100 %)	0.99	6 (50 %)	6 (50 %)	0.25	0 (0.0 %)	3 (100 %)	1.00	6 (67 %)	3 (33 %)	0.47
Palliative	4 (3 %)	152 (97 %)	0.70	42 (27 %)	114 (73 %)	0.12	14 (14 %)	88 (86 %)	1.00	28 (52 %)	26 (48 %)	0.79
Bridging for CAR	0 (0 %)	8 (100 %)	0.99	1 (13 %)	7 (87 %)	0.45	0 (0.0 %)	0 (0.0 %)	n/a	1 (13 %)	7 (87 %)	0.07
T-cell therapy												
Unknown	0 (0 %)	2 (100 %)	1.00	0 (0 %)	2 (100 %)	0.86	0 (0 %)	0 (0.0 %)	n/a	0 (0 %)	3 (100 %)	0.48

**Table 5:** Characteristics of patients with grade ≥ 3 toxicity.

Patient	Toxicity Grade	Toxicity	Diagnosis	Dose (Gy) /Fx	Treatment Site	CTV volume (cc)	RT technique	RT details	ST details
1	3	acute dermatitis	mycosis fungoides	12/3	extremity	n/a	electrons	electrons with bolus	Concurrent high dose methotrexate, >3 prior STs
2	3	acute dermatitis	DLBCL	30/6	extremity	719	photons	helical tomotherapy	RCHOP prior, ≤3 prior STs
3	3	acute pain	DLBCL	20/5	pelvis	n/a	photons		RGCVP prior, ≤3 prior STs
4	4	acute hematologic	leukemia	12/3	CNS	1959	photons	craniospinal irradiation	Cytarabine prior, ≤3 prior STs

Gy, Gray; Fx, fraction; CTV, clinical target volume; cc, cubic centimeters; DLBCL, diffuse large B cell lymphoma; RCHOP, rituximab/cyclophosphamide/doxorubicin/vincristine/prednisone; RGCVP, rituximab/gemcitabine/cyclophosphamide/vincristine/prednisone; CNS, central nervous system; ST, systemic therapy; n/a, not available.
